# Précis Elémentaire de Physiologie

**Published:** 1834-01-01

**Authors:** 


*Précis Elémentaire de Physiologie.* Par F. MAGENDIE. Troisième Edition. Deux Tomes.—Paris, 1833. 8vo. pp. 1084.

THE work before us is so well known as to render any analysis of its general contents unnecessary; nor, were this otherwise, would it be an easy task to give an abstract of it, since, from the multiplicity of subjects it embraces, and the admirably clear and simple style in which it is written, it scarcely admits of condensation. We have, however, selected it as the subject of an article, because we gladly avail ourselves of an opportunity of discoursing on matters of so much interest; because several parts of this work leave room, perhaps, for further illustration; and because, notwithstanding its general excellence, it contains some doctrines which appear to us so dissonant from the spirit of sound philosophy, and so much calculated to retard the progress of science, that we should wish to guard the student of physiology against adopting them too implicitly. We proceed therefore to make some remarks on a few of the many subjects offered for our consideration.

*Preliminary Views.* In his introductory observations on the general doctrine of life, our author shews himself strongly opposed to the hypothesis of a vital principle; and, if he had merely maintained that we have no absolute proof of the existence of any such principle, and that it ought not, consequently, to be made the basis of any physiological reasoning, we should have been disposed to agree with him; but, when he takes upon him peremptorily to deny its existence, he exceeds the limits of just reasoning, which admits as little of an arbitrary negation as of an unfounded assertion; and when he would refer all the phenomena of life to the agency of chemical and mechanical causes, (an opinion which, if not broadly expressed, is plainly to be inferred from the general tenor of his work,) it is obvious that he lends himself to an hypothesis just as unsupported by any kind of evidence as that which he would have us discard. No hypothesis, however specious, ought to be allowed to dictate the conclusions of the physiologist; but, if we had to choose between these two, we should not hesitate for a moment which to prefer. The matter stands simply thus: The believer in a vital principle observes a vast and wonderful series of phenomena, which he cannot explain by the application of any of those powers or properties of matter with which he is acquainted; he therefore, naturally enough, refers them to some power or property with which he is unacquainted, and to which, as to many other unknown things, he gives a name. The supporter of the opposite hypothesis, unwilling to admit the existence of anything which is not cognizable to his senses, has recourse to the laws of chemistry and mechanics to account for phenomena to whose solution they have hitherto, in every instance, been found utterly inadequate. We have no objection to see the hypothesis of a vital principle relinquished, because, though highly probable in itself, and highly useful and beautiful as a source of illustration, it ought never to have been maintained as a ground of reasoning; but we have a very great objection to see its place supplied by another hypothesis not at all better supported by facts, and, to our apprehension at least, infinitely less plausible. It is the excellent advice of M. Magendie to the student of medicine, early to accustom himself to say *I do not know:* now this is precisely what M. Magendie ought himself to have said with reference to vitality. A muscular fibre may contract when stimulated, owing to a peculiar power or property inherent in living matter; it may contract by means of chemical or mechanical adjustments, so intricate as to elude our present means of research; or, lastly, it may contract for no other reason than that it is the will of the Deity that it should contract. We are in a state of profound ignorance on the subject, nor is it very probable that we shall soon be much more enlightened. So powerfully, however, is the mind of M. Magendie possessed with his hypothesis, that he breaks forth, at the conclusion of the preface, into the following highly—*poetical* passage:

"Yet a few years, and physiology, intimately connected with the physical sciences, will not be able to make a step without their aid; she will acquire the rigour of their method, the precision of their language, and the certainty of their results; by thus raising herself, she will get above the reach of that ignorant and vain-glorious crowd, who always muster strong when the object is to oppose truth or imbibe error. Medicine, which is nothing but the physiology of the sick man, will speedily follow in the same path, and attain the same elevation; we shall thus witness the disappearance of those false explanations, the aliment of minds of the lowest order, which have so long disfigured her."

Truly, if this came to us under a less respectable name than that of Magendie, we should feel inclined to exclaim

"Parce puer stimulis, et fortius utere loris!"

We sincerely hope it may all come to pass, though we have our doubts; but of one thing we are very sure—that if these great results are to be attained, it must be by means of a philosophy more rigorous than the French professor has brought to his aid in the investigation of the vital powers.

We would not for a moment be supposed to undervalue the labours of the natural philosopher in behalf of the science of life; their importance must be universally felt and acknowledged; nor can we precisely understand our author's meaning when he says "the pernicious and absurd belief that physical laws have no influence on living bodies has no longer the same force." We really are not aware that any such opinion ever existed; we are certain that it does not exist at present. Our sole object in these remarks is to intreat physiologists not to travel too fast, lest they entail on their successors the irksome and thankless labour of unteaching all that they have taught; to remember that science, like ambition, may vault, and overleap itself; and not to lose sight of that short but most significant saying of the father of our art, ἡ τέχνη μακρή.

*Sensation.* That portion of the work which is devoted to sensation and the organs of sense has received some interesting additions: it is, however, to be regretted that the author has passed over in silence that *muscular sense*, which, although the name may be objected to by some, still unquestionably exists; nor is there any one sense on which the whole frame is more dependent for its preservation, or to which the other senses are more largely indebted for the correctness of their perceptions.

A moderate attention to the movements of our own bodies will be sufficient to convince us that we possess a power of estimating the degree and duration of muscular action, independently of the exercise of vision. Thus, when a man extends his arm while his eyes are shut, he is perfectly conscious of doing so, and knows whether the action is performed gently or forcibly, rapidly or slowly. This may indeed depend on a mere modification of touch, by which the muscular fibres are sensible of each other's pressure in the act of contraction; but it more probably arises from a distinct sense, peculiar to the voluntary muscles. We think it possible, also, that our estimate of the degree of muscular action may be partly formed, independently of this sense, from the consciousness of the intensity of volition necessary to produce a given effect upon the muscles.

Our knowledge of the position of the body, and the adjustment of muscles necessary to preserve its balance, and to effect its innumerable changes of posture and locality, all depend on the exercise of this sense, conjointly with that of vision: that the latter, however, acts the least important part in these functions is proved by the fact that blind persons have a very sufficient command over their muscles, and even acquire, by practice, a more perfect control over some of their motions than is generally possessed by those who can see.

It is singular that this admirable sense should never have fairly attracted the attention of physiologists, till it was pointed out, and very ingeniously applied, by Dr. Thomas Brown, the late distinguished professor of moral philosophy in the university of Edinburgh; whose name, however, we have seldom seen mentioned in connexion with it. (*Lectures on the Philosophy of the Human Mind,* vol. i. p. 496.) This author shews that a number of those physical properties of bodies which are supposed to be ascertained merely by the touch, are, in fact, made known to us through our notions of *resistance,* and that our idea of resistance is derived entirely from the estimate of muscular action.

"By touch we are commonly said to be made acquainted with extension, magnitude, divisibility, figure, motion, solidity, liquidity, viscidity, hardness, softness, roughness, smoothness. These terms I readily allow are very convenient for expressing notions of certain forms or states of bodies that are easily distinguishable. But, though specifically distinguishable, they admit generically of very considerable reduction and simplification. Hardness and softness, for example, are expressive only of greater or less resistance; roughness is *irregularity* of resistance, when there are intervals between the points that resist, or when some of these points project beyond others; smoothness is complete uniformity of resistance; liquidity, viscidity, are expressive of certain degrees of yieldingness to our effort, which solidity excludes, unless when the effort employed is violent. All, in short, I repeat, are only different species or degrees of that which we term resistance, whatever it may be which impedes our continued effort, and impedes it variously, as the substances without are themselves various." (*Op. citat.* vol. i. p. 487.)

Our notion of *weight* is no doubt originally derived from the same source, being formed on an estimate of the resistance offered to muscular action by the tendency of bodies to descend; or, in other words, of the degree of muscular exertion necessary to sustain them.

This subject of the muscular sense admits also of a very interesting application to the theory of vision. In treating of this sense, M. Magendie has not made any direct allusion to the curious subject of erect vision from an inverted image on the retina; but he introduces the hypothesis on which the fact is now most frequently explained in the following words:

"By the effect of an admirable instinct, we know the direction of the light which penetrates the retina; it appears that we establish in principle that light moves in a right line, and that this line is the prolongation of that which the light followed before penetrating the cornea."

The opinion here advanced is nearly the same with that given by Sir D. Brewster; who, however, has expressed it more distinctly thus:

"We know nothing more than that the mind, residing as it were in every point of the retina, refers the impression made upon it at each point to a direction coinciding with the last portion of the ray which conveys the impression." (*Edinburgh Encyclopædia*, art. *Optics*.)

Now we think that, on close inspection, this solution will be found insufficient, and for the following reasons. The mind cannot perceive the direction of the ray of light, since it does not perceive the ray itself: a thing cannot be at once the means and the object of perception; and we learn from science, not from sensation, that there are any rays of light proceeding from the external object of vision to the eye. It is not, then, the ray which is perceived, but the impression made by that ray; and, to talk of the direction of an impression, would be to use words without a meaning. If, however, the sentence just quoted is intended merely to signify that the impression made on the lowest point of the retina is, somehow or other, known to be derived from the highest point of the object, and *vice versâ*, the whole is nothing more than a circuitous mode of stating the fact, that, though the image on the retina is inverted, the object is nevertheless seen in its real position; which is the very thing to be accounted for.

We advance this with great submission, being well aware of the weight of the authority from which we have ventured to dissent.

This theory, moreover, as well as some others less worthy of notice, rests on the assumption that the image depicted on the retina is the direct object of the mind's perception; of which we have no proof. It is therefore well remarked by Dr. Bostock, that there seems "no reason why the inversion of the image should lead to the conception of an inverted object, rather than the contrary; and hence the question that has been so frequently asked, why do we not see objects inverted? may be answered by asking, in return, why should we expect this to be the case?"

It appears to us that by far the happiest solution of this difficulty hitherto offered, is that which Sir C. Bell has framed, with much ingenuity, on the doctrine of the muscular sense. "There is," says this accomplished physiologist, "an inseparable connexion between the exercise of the sense of vision and the exercise of the voluntary muscles of the eye. When an object is seen, we enjoy two senses: there is an impression upon the retina, but we also receive the idea of position or relation, which it is not the office of the retina to give. It is by the consciousness of the degree of effort put upon the voluntary muscles, that we know the relative position of an object to ourselves." *(Phil. Trans. for* 1823.) According to this theory, then, we are enabled to distinguish whether the entire object be above or below us, to the right hand or to the left, by a consciousness of the action of that particular muscle which directs the eye towards the object. In like manner, we are enabled to distinguish the top of an object from the bottom, by a consciousness of the action of the levator muscle when we regard the former, and of the depressor when we regard the latter. We have heard it ingeniously objected to this view of the subject, that it will not apply to objects so minute as to require no muscular action to enable the eye to apprehend them, but which are nevertheless seen in their true position as correctly as larger objects. This objection, however, is not valid, because, although no muscular action is required to enable the eye to traverse the surface of such objects, yet the smallest elevation of the eye banishes the object, and the point which disappears last is thus known to be the top: in like manner, the smallest depression of the eye also banishes the object, and the point which disappears last is known to be the bottom; or, in more general terms, we ascertain its position by an instantaneous act of comparison with the objects already known to be above or below it.

We may extend this view of the action of the muscles of the orbit to the explanation of our notion of *magnitude.* On this subject, our author remarks, that our judgment of the dimensions of bodies depends much more on intelligence and habit, than on the actual apparatus of vision; and that we acquire correct ideas on this head, from the size of the image formed on the retina; the intensity of the light which emanates from the object; the distance at which we believe it to be placed; and, above all, from the habit of comparing objects, when seen together. We think it must be at once apparent that these circumstances, although they have considerable influence, are not alone sufficient to account for our perception of the magnitude of bodies; but if we call to their aid the action of the muscles of the eye, our explanation becomes much more satisfactory. It is, we think, highly probable that our estimate of the magnitude of bodies depends, in a great measure, on a consciousness of the extent and duration of muscular action requisite to carry the eye over their entire surface, in its several dimensions; and, with reference to objects of extreme minuteness, on the consciousness that no such action is required, and that the smallest movement of the eye diverts it entirely from the object. Our judgment is no doubt much aided by comparison, and by our estimate of the distance of the object, which we form from the necessary adaptation of the axes of the two eyes, the distinctness and colour of the object, the number of intervening objects, and perhaps some other considerations.

We trust we have said enough to show that the subject of the muscular sense ought not to be neglected.

Before quitting altogether the subject of the senses, we wish to advert to a point which, we believe, has not hitherto attracted the attention of physiologists, or, at least, not so much attention as it may perhaps deserve. We have been accustomed to consider the impressions made on the nerves of sense solely as a means of the mind's converse with the external world; but, is it not possible that they may also have a sympathetic influence, as stimuli, on the whole nervous system, and, consequently, on the whole frame; thus forming a part, though a very subordinate one, of the general impulse by which the vital actions are carried on?

Take for example the sense of sight. It is well known that a continued exposure to a strong glare of light will produce headach and dizziness; and the observations of M. Andral render it probable that the supposed influence of the moon on lunatics arises entirely from the excitement produced by her light. On the other hand, those accustomed to the treatment of nervous patients, must have frequently observed their sudden improvement on being transferred from a gloomy to a lighter apartment, and the disappearance of their daily symptoms of languor and oppression on the introduction of artificial light in the evening: perhaps, however, a cheerful influence on the mind may have much to do with these effects.

Again, with respect to the sense of hearing. The effect of continued noise on those whose nervous system is naturally irritable, is so great as to induce general discomfort, restlessness, headach, and even a degree of febrile excitement. Dr. Mead, who in his Essay on the Tarantula has some remarks on this subject, gives the following instance of the powerful effect of sound on a dog; not, however, on his own authority, though on that of an eye-witness. "A player on a fiddle, having frequently observed that a dog in the room was always so affected by a particular note as to howl and show great uneasiness at it, one day, for experiment's sake, continued to strike the same note so long, till the sensible animal fell into convulsions, and died." (*Medical Works*, p. 77.)

With reference to the sense of smell, it is well known that many Roman women, and even men, will faint, or become hysterical, at the fragrance of a rose. Now, is it not probable, from these, and similar facts, of which a great number might be adduced, that, as inordinate impressions on the nerves of sense produce such strong effects on the entire system, the ordinary and moderate impressions to which they are continually subjected must also produce a certain degree of effect, and may perhaps form a part of the necessary stimuli of the nervous system, and, through it, of the healthful actions of life? We merely throw out the suggestion in a very crude form, apologizing for introducing it where it may appear to be irrelevant; but the prosecution of this subject might perhaps reward the labours of the physiologist, and also throw some light on the phenomena of disease.

*Functions of the Brain.* When we reflect that this subject has afforded constant occupation to philosophers of all ages and nations since the days of Aristotle, and that we know about as much of the matter now as was known to the Stagyrite, it is with no small degree of astonishment we learn from M. Magendie, that this study "is perhaps easier than that of most other functions." Let us hear what our author believes these functions to be.

"The most wonderful and sublime developments of human nature, intelligence, thought, instinct, the passions, and that admirable faculty by which we direct our movements and exercise speech, &c. are phenomena so dependent upon the brain, that many physiologists designate them by the appellation of *cerebral functions*. Other physiologists, sustained and inspired by religious faith, regard them as appertaining to the soul, a being of a divine essence, one of whose attributes is immortality."

M. Magendie remarks, with great propriety, that the physiologist need not involve himself in theological disputes, as the one subject has nothing to do with the other. Now, if we understand this passage aright, our author asserts that intelligence and thought depend upon the brain;—are functions of the brain; at least, that the only ground on which this doctrine can be questioned is that of religion.

We would, however, suggest a very different ground; simply, that there is not a single unequivocal fact in its favour, and that all reasoning is against it. It has indeed been adopted by many physiologists; but the whole history of science does not present an example of a more gratuitous assumption. Observation has taught us that the brain is the general receptacle of all impressions made on the external senses, and the centre from which volition is communicated to the organs subservient to the will: we know no more. The facts so often appealed to by the materialist of the correspondence of the mental phenomena with the development of the brain in man and the higher animals, and of the derangement of those phenomena by disease of the brain, are really quite irrelevant, and prove nothing on one side or the other; because, supposing the brain to be merely the medium that conveys to the mind those impressions which all acknowledge to be its necessary food, and the foundation on which it builds all its operations, it follows, as a matter of course, that the more perfectly the brain is organized, the more perfect and numerous those impressions will be, the more ample the materials of thought, and the more complete the development of the mind. Again, when those impressions are rendered imperfect and confused by disease of the organ on which they depend, it follows, as a matter of course, that the mind cannot work properly on such insufficient materials.

M. Magendie may *conjecture* that the brain is the organ of thought; but, for our part, when we consider that thoughts are things which geometry cannot measure, which chemistry cannot analyse, which accumulate without filling space, and are incessantly active without motion; when, in short, we cannot trace in them the remotest analogy to any material product or result, our conjectures are strong against referring them to the action of any material organ whatsoever. Nevertheless, we freely disclaim all right to deny that it may be as M. Magendie says; but, in assuming that it is so, he substitutes opinion for fact. Truth obliges us to confess that we know no more about the matter than the dead brain whose structure we unravel, and on whose functions we speculate. The phrenologists will not be pleased with us for saying this, since they profess to bring forward *facts* relative to this matter; but, although the spirit of observation by which the votaries of that science are actuated cannot be too much commended, we fear that, till their method of observing be changed, phrenology can never prove or disprove any thing. It sets out on a most unfortunate maxim, δεῖ μὲν τὸν μανθάνοντα πιστέυειν: we must begin by admitting the truth of the system, since no one organ can be established without previously taking several others for granted.

*Nervous System.* The important views of our author on this branch of physiology must now be familiar to every one, and they are for the most part too well established to leave room for any discussion. The part of the nervous system which most completely baffles the ingenuity of the physiologist is that called the ganglionic—the great sympathetic nerve—if indeed we have any right thus to designate it. From the frequent use made of the supposed functions of this nerve in framing pathological theories, we are apt to delude ourselves into a notion that we have actually some positive knowledge concerning it: the reverse, however, is the fact. On this subject M. Magendie has the following sensible note, slightly enlarged from the preceding edition.

"Is the great sympathetic a nerve? The ganglions and filaments which arise from it, or proceed to it, have no analogy with nerves properly so called: they differ entirely in colour, form, consistence, disposition, structure, properties of tissue, and chemical properties. Nor is there any greater analogy in their vital properties; a ganglion may be pricked, cut, or torn, without the animal appearing conscious of it, and without any contractions taking place in the muscles. I have often tried these experiments on the cervical ganglia of dogs and horses; similar operations, if performed on the sensible nerves of the brain, would have produced horrible torture; if on the insensible, or those of motion, powerful muscular contractions. Let all the cervical and the first thoracic ganglia be removed; we do not perceive any sensible and immediate derangement in the functions even of those parts to which their filaments are distributed. What reason then is there to consider the ganglionic system as forming part of the nervous? Would it not be wiser, and more conducive to the future progress of physiology, to admit that the use of the great sympathetic is at present entirely unknown? One is confirmed in this idea by the perusal of authors. One sees, for instance, the ganglions considered as nervous centres, as little brains, as nuclei of grey matter destined to nourish the nerves, &c. If the proofs are required on which these authors establish their doctrine, we are astonished to find that there are none, and that their assertion is nothing but a *jeu d'esprit*. After the unproductive inquiries which have been instituted up to the present day, it appears to me the most probable conjecture with respect to this singular organ, so intimately connected with the nerves, that its functions are of a peculiar order, to which physiologists have not yet obtained the clue, but which may reveal itself to him who knows how to interrogate nature by delicate and ingenious experiments."

Comparative anatomy is the source from which we are most likely to obtain information on this, and other organs whose functions are unknown. The system of animated nature is indeed one vast physiological museum, in which we see parts developed and displayed in the order in which their functions are required; and curtailed, or removed, not by the knife of the anatomist, which extinguishes vitality while it exhibits structure, but by the working of a power which obliterates each part as its function becomes useless, still preserving uninjured the mechanism of life.

The chief obstacle in the way of applying this mode of investigation to the ganglionic system, consists in the difficulty of ascertaining at what point in the animal scale it actually commences; it being doubtful whether the ganglia which make their first appearance in the Mollusca, correspond with those of the spinal nerves, or those of the sympathetic, or are different from either. Further observation will, however, most probably clear this matter up.

After all, the apparently universal distribution of the sympathetic to all the organs of the body, and its connexion with every part of the nervous system, afford much reason for believing that it in some manner presides over and controls all the actions of organic life, and associates them with each other, and with the animal functions.

*Animal Heat.* As no new experiments have been made which can be considered as conclusive with regard to the source of animal heat, and as all the principal theories on the subject, with the experiments on which they rest, are no doubt abundantly familiar to our readers, we should not here have adverted to it, had we not wished to direct attention to a very singular pathological phenomenon, which we think goes far to overturn the chemical theory. M. Magendie professes himself a believer in this doctrine, more decidedly, perhaps, than the facts of the case warrant. It must, however, be allowed, that it is very strongly supported by numerous and striking experiments; by the fact, that much heat, however it may be disposed of, must unquestionably be generated by the chemical process continually going on in the lungs: and by the parallel which comparative anatomy enables us to draw between the perfection of the respiratory function, and the degree of animal temperature; it being pretty universally found, that the more the lungs, by their extent and organization, favour the chemical changes in the blood, the higher is the temperature of the animal in proportion to the medium in which it lives. Beautiful however as this theory is, and sorry as we should feel to be obliged to give it up, we cannot help remarking, that a phenomenon very difficult to be reconciled with it presented itself frequently in the course of the late extraordinary epidemic called the *cholera.* We have ourselves observed, not once, but repeatedly, and the observation has been made to us by others, that when this disease was within a few minutes of its fatal termination, when the surface had for hours been cold, and the pulse entirely extinct, and when respiration was reduced to a feeble attempt to dilate the chest, made at long intervals, the heat of the surface has sometimes suddenly returned, so that at the moment of death, and for a short time after, it has been little, if at all, below the natural standard. Now in this case no heat can either be developed in the lungs, or become latent in the blood, by the process of arterialization, because that process does not take place, the blood being as black as pitch; nor can any heat be evolved on the surface of the body, by the change of arterial into venous blood, because the circulation through the minute vessels does not go on; and, if it did, there is no arterialized blood to be changed. To the fact here stated we can testify, on our own personal experience; indeed, it probably attracted general attention. All this throws no light on what *is* the source of animal heat; but it shows, we think, pretty clearly that respiration *is not;* at all events, that it is not the only, nor the principal source.

There are many other subjects in these interesting volumes on which we could willingly dilate; but, lest the reader should say *claudite jam rivos,* we will do it of our own accord.

